# Effect of Fucoidan on Anterior Cruciate Ligament Transection and Medial Meniscectomy Induced Osteoarthritis in High-Fat Diet-Induced Obese Rats

**DOI:** 10.3390/nu10060686

**Published:** 2018-05-28

**Authors:** Sabri Sudirman, Alan Darmasaputra Ong, Heng-Wei Chang, Zwe-Ling Kong

**Affiliations:** Department of Food Science, National Taiwan Ocean University, 202 Keelung, Taiwan; sabrisudirman@unsri.ac.id (S.S.); spillidan@gmail.com (A.D.O.); romeo5566boy@gmail.com (H.-W.C.)

**Keywords:** anterior cruciate ligament, fucoidan, meniscectomy, obesity, osteoarthritis

## Abstract

Osteoarthritis (OA) has become one of the most common disabilities among elders, especially in females. Obesity and mechanical injuries caused by OA are attributed to joint loading, cartilage disintegration, and bone loss, as well as inflammation. Pharmacological and non-pharmacological treatments can be used for OA. Fucoidan possesses several bioactivities such as antitumor, antiviral, anticoagulation, anti-obesity, and immunomodulation. This study aims to investigate the effect of fucoidan in surgery-induced OA on rats with diet-induced obesity. OA was induced by an anterior cruciate ligament transection and a partial medial meniscectomy (ACLT + MMx). The male SD rats were fed with a high-fat diet (HFD) for 4 weeks to induce obesity before causing ACLT + MMx to induce OA. The OA rats were administered with intragastric water or fucoidan in three different concentrations (32 mg/kg, 64 mg/kg, and 320 mg/kg) after the surgeries for 40 days with an HFD. We observed that the swelling in the knee joint was alleviated and the hind paw weight distribution was rectified after feeding them with fucoidan and that there was no significant effect on the weight gain and feed intake. Fucoidan administration indicated no significant variation on the high-density lipoprotein (HDL)-Cholesterol level, but it did indicate reduced plasma triglycerides and low-density lipoprotein (LDL)-Cholesterol levels. In addition, the weight-bearing tests showed an improvement in the fucoidan-treated group. Our results suggested that fucoidan may improve meniscal/ligamentous injury and obesity-induced OA.

## 1. Introduction

Osteoarthritis (OA) is the most prevalent form of arthritis [1]. OA is one of the most common chronic health conditions and a leading cause of disability and pain among adults [2]. OA has an inflammatory component affecting the synovium and cartilage, which leads to subchondral bone tissue breakdown, resulting in pain, stiffness, and joint failure [3,4,5]. Several studies have suggested that OA joint degeneration occurs from a combination of mechanical stresses and biochemical factors [4,6]. Chondrocytes as well as synovial cells, in an elevated OA condition, express inflammatory cytokines such as interleukin (IL)-1β and the tumor necrosis factor (TNF)-α. As a result, there is an increase in the matrix metalloproteinase (MMPs) and some pro-inflammatory cytokines such as IL-8, IL-6, prostaglandin E2, and nitric oxide [5].

OA models can be classified into primary (spontaneous) and secondary (post-traumatic OA, including induced models) OA [7]. Obesity is evaluated as a worldwide health concern with low-grade inflammatory status [8]. Obesity has long been recognized as a potential risk factor for OA, especially of knee OA [9]. By increasing the mechanical forces across weight-bearing joints, obesity or the excess of body weight may lead to cartilage degeneration [10,11]. Other causes such as inflammation and lipid metabolism disorder in obesity are also associated with OA [12]. Recent studies reported that inflammatory cytokines such as leptin, adiponectin, and IL-1β are involved in obesity-associated OA progression [13,14]. Anterior cruciate ligament (ACL) tears are commonly correlated with the impairment of the articular cartilage, menisci, subchondral bone, and other ligaments [15,16,17]. It is believed that around 50% of acute ACL tears are accompanied by a meniscal injury, while in the chronic ACL-deficient knees, as high as 80% of the patient population have been observed to have meniscal tears [15,18]. The most critical risk factor for developing knee osteoarthritis after an ACL injury might be meniscectomy [19]. Previous studies reported that the obesity model from a high fat/high sucrose diet is an independent risk factor for the onset of OA in rats with anterior cruciate ligament-transected (ACL-X) knees [20]. Furthermore, serum and synovial fluid may be involved in OA progression through the regulation of pro-inflammatory markers such as IL-1β, IL-6, IP-10, and leptin in obesity conditions [21]. In addition, several pathways that lead to musculoskeletal complications such as muscle complication, bone, tendons, and joints are caused by metabolic syndromes (that is, visceral obesity, hypertension, and dyslipidemia) [22].

Various strategies used for the management of OA include non-pharmacological and pharmacological treatments. Pharmacological treatments include analgesics or anti-inflammatory agents such as acetaminophen, cyclooxygenase (COX)-2 inhibitors, glucosamine/chondroitin sulfates, non-selective non-steroidal anti-inflammatory drugs (NSAIDs), and intra-articular (IA) corticosteroids. NSAIDs are correlated with an increased risk of cardiovascular (CV), severe gastrointestinal (GI), and renal injuries [23,24,25]. Based on this condition, various studies have focused on functional foods for the treatment OA, which may result in the promotion of cartilage health and safety, even after long-term use. 

Fucoidan is a sulfated polysaccharide that contains *L*-fucose and sulfate groups and found in various species of brown seaweed such as *Sargassum binderi* [26], *Undaria pinnatifida* [27], *Fucus vesiculosus* [28], *Laminaria japonica*, and *Hizikia fusiforme* [6]. Because of their pharmacological properties such as the antioxidant, anti-tumor, anti-inflammation, anti-diabetic, and anti-obesity properties, fucoidan has gained significant attraction [26,27,29]. Recent studies indicated that fucoidan has the potential to suppress inflammation in collagen-induced arthritis [30]. Furthermore, fucoidan is potent as a therapeutic agent [31]. This study was aimed to investigate the hypolipidemic and anti-inflammatory properties of fucoidan. Moreover, it was aims to determine the effects of fucoidan on rats fed with a high-fat diet (HFD) with anterior cruciate ligament transection (ACLT) and medial meniscectomy (MMx) surgery induced OA.

## 2. Materials and Methods

### 2.1. Fucoidan

The fucoidan (low-molecular-weight (MW) ~5000 Daltons) was prepared from *Cladosiphon okamuranus* by hot water extraction and enzymatic digestion, provided by Simpson Biotech Co., Ltd. (Taoyuan, Taiwan).

### 2.2. Animal Model

**Figure 1:**
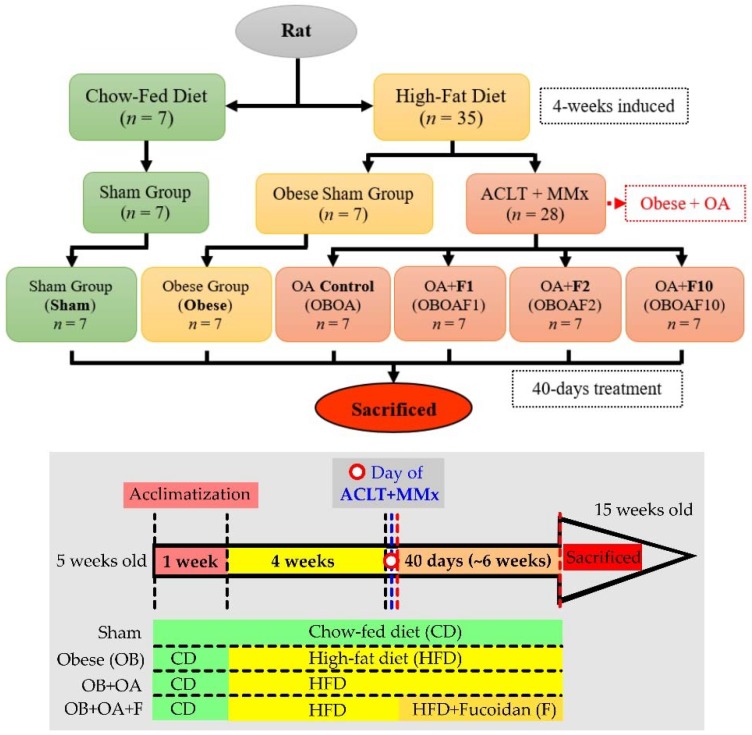
The flowchart of high-fat diet-induced and an anterior cruciate ligament transection and a partial medial meniscectomy (ACLT + MMx) surgery on an osteoarthritis rat model.

All of the procedures were carried out according to the Animal Protection Act (Act/APC) and the Experimental Animal Ethics Committee of the Council of Agriculture (CoA) of the Executive Yuan, Taiwan. The Institutional Animal Care and Use Committee (IACUC Approval No. 107003) of the National Taiwan Ocean University reviewed and approved all protocols. Five-week-old male Sprague Dawley (SD) rats were purchased from BioLASCO Taiwan Co., Ltd. (Yilan, Taiwan). The rats were housed individually in cages in an animal room with a 12 h light/dark cycle at a temperature of 25 ± 2 °C and 55% humidity. During the experiment, the diet foods and water were provided ad libitum. During the acclimatization phase (1 week), all the rats were given a chow-fed diet (LabDiet^®^ 5001 Rodent Diet, composed of 13.38% kcal from fat, 57.95% kcal from carbohydrates, and 28.67% kcal from protein). After acclimatization, the rats were divided into 2 main groups, the Sham and Obese group. The obese group was given a high-fat diet (HFD, composed by 45.00% kcal from fat, 36.04% kcal from carbohydrates, and 18.97% kcal from protein) for 4 weeks to develop obesity (approximate body weight > 20% of the ideal/sham weight) as described by previous studies [32] and continued until the end of experiment (fed with a HFD for approximately 10 weeks). Following the HFD induction, the obese rats were divided into the obese sham (OBSham) group and the OA (OBOA) group. Anterior Cruciate Ligament Transection and Medial Meniscectomy (ACLT + MMx) surgeries were performed to induce OA (Figure 1). For this purpose, the rats were anesthetized with Zoletil 50 (25 mg/kg, intraperitoneal (i.p.)) and the hair on the right knee was clipped. An incision was made in the medial aspect of the joint capsule (anterior to the medial collateral ligament), the ACL was transected, and the medial meniscus was removed. After the surgery, the joint was irrigated with normal saline, the capsule was sutured with 4–0 chromic catgut, and the skin was closed with 4–0 silk braided sutures. In the sham-operated rats, incisions were made in the medial aspect of the joint capsule to expose the ACL, but the ACL was not transected nor was the medial meniscus removed. The rats were supplied with supplemental heat and were monitored until recovery from anesthesia. The rats were also checked daily regarding their general health and regarding pain, discomfort, and infection in the post-operative period. Cefazolin (20 mg/kg i.p.) was injected after the surgery to prevent infection. Following the surgery, the rats were intragastrically treated with different doses of fucoidan: doses of 32 mg/kg body weight (F1), 64 mg/kg (F2), or 320 mg/kg (F10) daily for 40 days. Their body weights were measured weekly with a digital balance and the width of the knee joint was measured using digital calipers before the surgery and every week for 40 days after the surgery. Additionally, Incapacitance tests were performed weekly before and every week after the surgery within the duration of 40 days. The animals fasted for 12 h before sacrifice. The rats were sacrificed at the age of 15 weeks by exposure to CO_2_ until euthanasia in an empty chamber. The blood samples were collected and the operated knees were dissected after all tests were completed.

### 2.3. Measurement of Plasma Biochemical Parameters

Whole blood samples were centrifuged after collected at the day of sacrifice and the blood plasma were separated from the blood pellets. The plasma samples were preserved at −80 °C, ready for use. The plasma triglycerides (TG), total cholesterol (TC), high-density lipoprotein-cholesterol (HDL-C), low-density lipoprotein-cholesterol (LDL-C), superoxide dismutase (SOD), and glutathione peroxidase (GPX) were measured with commercial enzymatic kits (Randox, UK). The tumor necrosis factor-α (TNF-α), interleukin-1β (IL-1β), and adipokine (leptin) were measured with the ELISA kit (Abcam, Cambridge, UK; R & D Systems, Minneapolis, MN, USA.; Novex Life Technologies, Waltham, MA, USA, respectively).

### 2.4. Weight-Bearing Distribution Assessment

The weight-bearing distribution changes were measured using an Incapacitance tester (Linton Instrumentation, Norfolk, UK) to detect changes in the postural balance based on previous methods [33]. In particular, the rats were stood on their hind paws in an inclined plane (65° from horizontal) chamber that was placed above the incapacitance apparatus; the weight applied to each hind limb was measured independently with the apparatus. Three to five measurements, each of them being 5-s readings, were taken for each rat, and the average was calculated after excluding outliers. The data were expressed as the difference between the weight applied to the limb contralateral to the injury and the weight applied to the ipsilateral limb (Δ Force).

### 2.5. Knee Width and Joint Histopathology

The width of the knee joint was measured with digital calipers every week for 40 days after the operation and the width of the contralateral knee was used as the baseline. At day 40, after all the tests were completed, the rats were euthanized with carbon dioxide and the knee joints were collected and fixed in 4% paraformaldehyde for 2 days. The decalcification, embedment in paraffin, and histological sectioning (5 mm) that followed were done by Li Pei Co. Ltd. Hematoxylin/eosin (H&E) staining and Safranin-O staining were then used to examine the morphological changes and proteoglycan loss. The pathology of the OA cartilage was graded according to the Osteoarthritis Research Society International from grades 0–6 (OARSI grades 0–6) based on the previous method [34,35]. Normal cartilage has a grading of 0 (hyaline articular cartilage uninvolved with OA) and the following six grades depended on the severity of OA. Grade 1 is the threshold in cartilage for OA and characterized by the retention of the articular cartilage surface layer; Grade 2 is characterized by focal discontinuity of the cartilage superficial zone; Grade 3 is the extension of matrix cracks into the mid zone to form vertical fissures (clefts); Grade 4 is cartilage erosion; Grade 5 is characterized by denudation, the complete erosion of the hyaline cartilage to a level of mineralized cartilage or bone; and Grade 6 is recognized by changes in the contour of the cartilage surface (deformation).

### 2.6. Statistical Analysis

All experimental data are expressed as mean ± standard error of mean (S.E.M.). The body weight, weight-bearing difference, and knee width were analyzed with a Two-way analysis of variance (Two-way ANOVA) followed by Dunnett’s test. The other variables were analyzed with a one-way ANOVA followed by Duncan’s multiple comparison tests with *p* < 0.05 defined as statistically significant.

## 3. Results

### 3.1. Reduction of Body Weight and Body Lipid by Fucoidan

The body weights of HFD-induced obese rats were significantly increased compared to the sham group (*p* < 0.05). After treating them with fucoidan for 40 days, the body weights were lowered by 9%. The perirenal adipose tissue weight also decreased after fucoidan treatments (Table 1). The TG, TC, and LDL-C levels of rats fed with the HFD were significantly (*p* < 0.05) higher before undergoing treatment with fucoidan (Table 2).

### 3.2. The Effect of Fucoidan on the Antioxidant Properties and Anti-Inflammatory Properties

**Figure 2:**
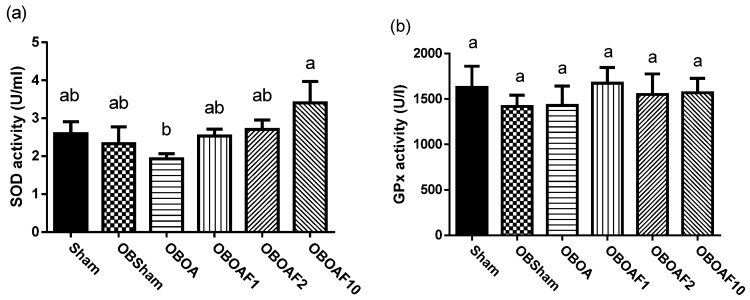

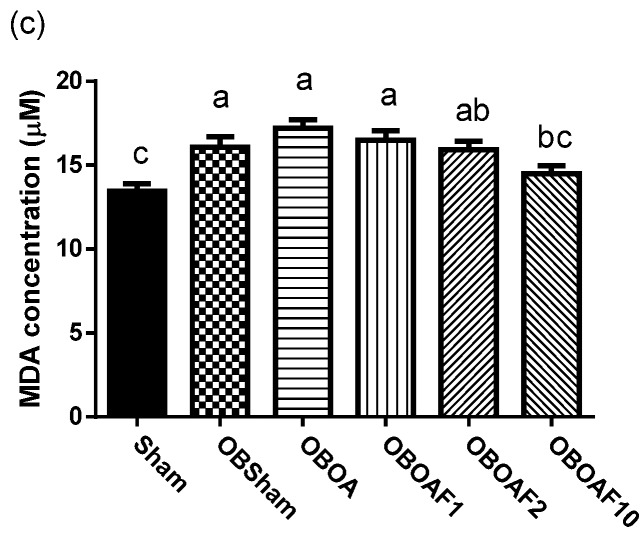
The effect of fucoidan treatment on the antioxidant activities in HFD-induced obese male rats with ACLT + MMx surgery induced OA. (**a**) Superoxide dismutase, SOD. (**b**) Glutathione peroxidase, GPx. (**c**) Malondialdehyde, MDA. The data are the activity of each enzyme and the concentration of the plasma reactive oxygen species, expressed as the mean ± S.E.M (*n* = 7). The values with different superscript letters (a)–(c) represent significant differences (*p* < 0.05) via one-way ANOVA followed by Duncan’s multiple range test.

**Figure 3:**
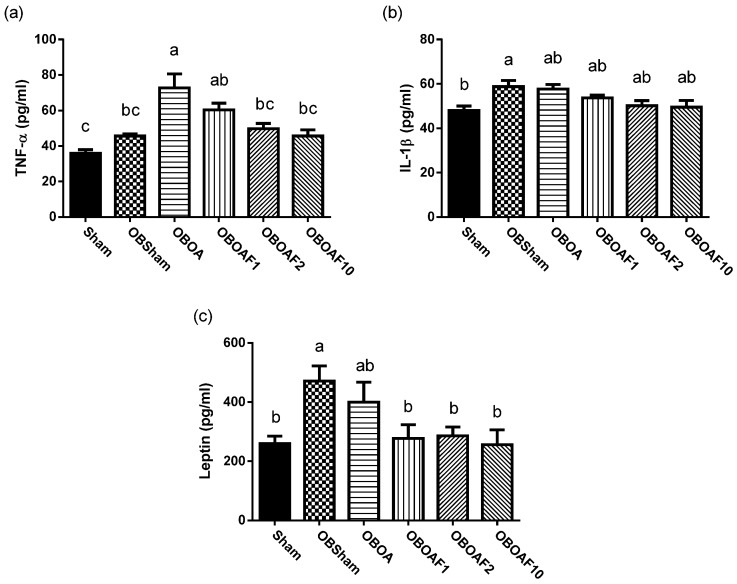
The effect of fucoidan treatment on plasma cytokines in HFD-induced obese male rats with ACLT + MMx surgery induced OA. (**a**) Plasma tumor necrosis factor (TNF)-α; (**b**) Interleukin (IL)-1β. (**c**) Leptin. The data are the concentrations of each cytokine, expressed as the mean ± S.E.M (*n* = 7). The values with different superscript letters, “a” and “b”, represent significant differences (*p* < 0.05) via one-way ANOVA followed by Duncan’s multiple range tests.

The antioxidant activity of SOD and GPx were decreased and the plasma MDA increased in the HFD fed groups. Treatment with fucoidan restored the activities of SOD and GPx and reduced the plasma MDA (Figure 2). There is an increase in the production of pro-inflammatory cytokines, mainly associated with the chronic systemic inflammation induced by obesity. In rats fed with the HFD, the plasma inflammatory cytokine was increased, especially TNF-α and leptin (Figure 3). Treatment with fucoidan reduces the inflammatory cytokines in plasma compared to the HFD fed untreated groups.

### 3.3. Effects Fucoidan on Joint Histology

**Figure 4:**
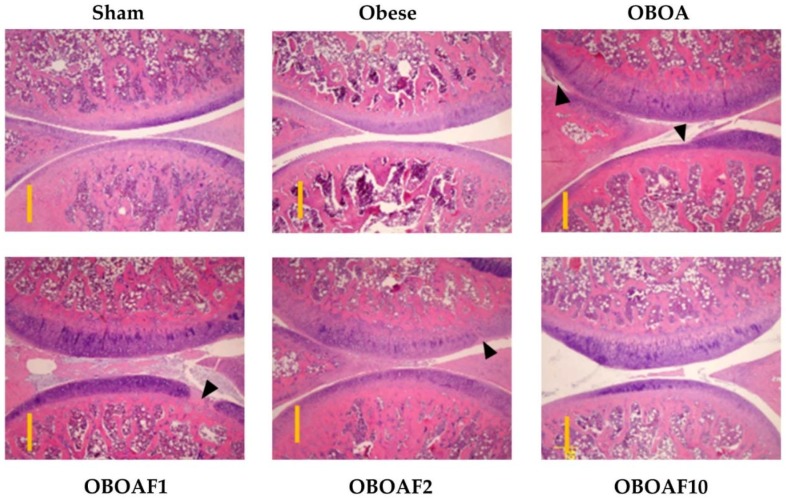
The histopathological difference between the knee joints in the HFD-induced obese male rats with ACLT + MMx surgery induced OA using Hematoxylin and Eosin stains. The representative cartilage sections from the right medial condyle of the femur and tibia were stained. The specimens were observed with 40× magnification. The scale bar length is 500 μm.

**Figure 5:**
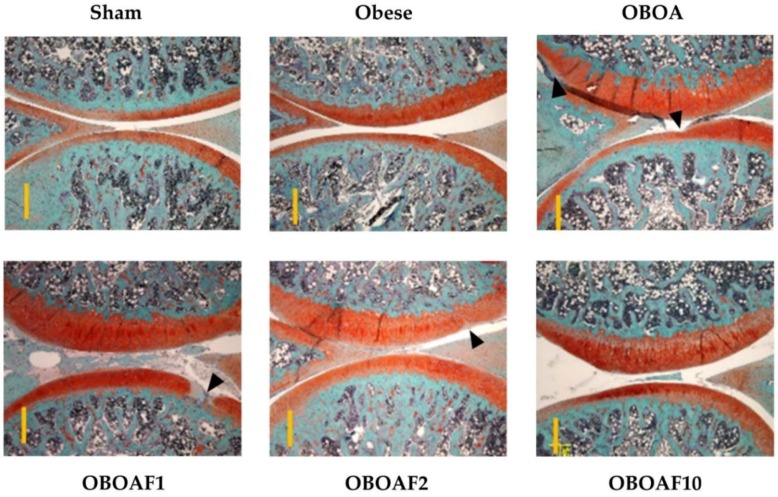
The histopathological difference between the knee joints in HFD-induced obese male rats with ACLT + MMx surgery induced OA using Fast Green and Safranin-O stains. The representative cartilage sections from the right medial condyle of the femur and tibia were stained. The specimens were observed with 40× magnification. The scale bar length is 500 μm.

At the end of the experiments, the rats were euthanized and the knee joint specimens were collected. The joint sections were stained with hematoxylin and eosin stain to observe the morphological changes by surgery-induced OA. The result showed a reduction of the cartilage thickness in the OBOA group while an improvement was observed in the fucoidan-treated groups (Figure 4). Other joint sections were stained with Safranin-O and fast green to observe the proteoglycan loss by OA. In the OBOA group, the joint histology showed a major loss of proteoglycan in the cartilage matrix. The treatment of fucoidan prevented further proteoglycan loss (Figure 5). As shown in Figure 4 and Figure 5, the sham group’s in articular cartilage was uninvolved with OA (grade 0). However, in the OBOA group as well as in the OAOBF1 group, cartilage erosion and denudation were seen (grades 4–5). The treatment with fucoidan in high concentration reduced the articular cartilage damage with the retention of the articular cartilage (grade 1).

### 3.4. Fucoidan Attenuate OA Caused Pain and Damage

**Figure 6:**
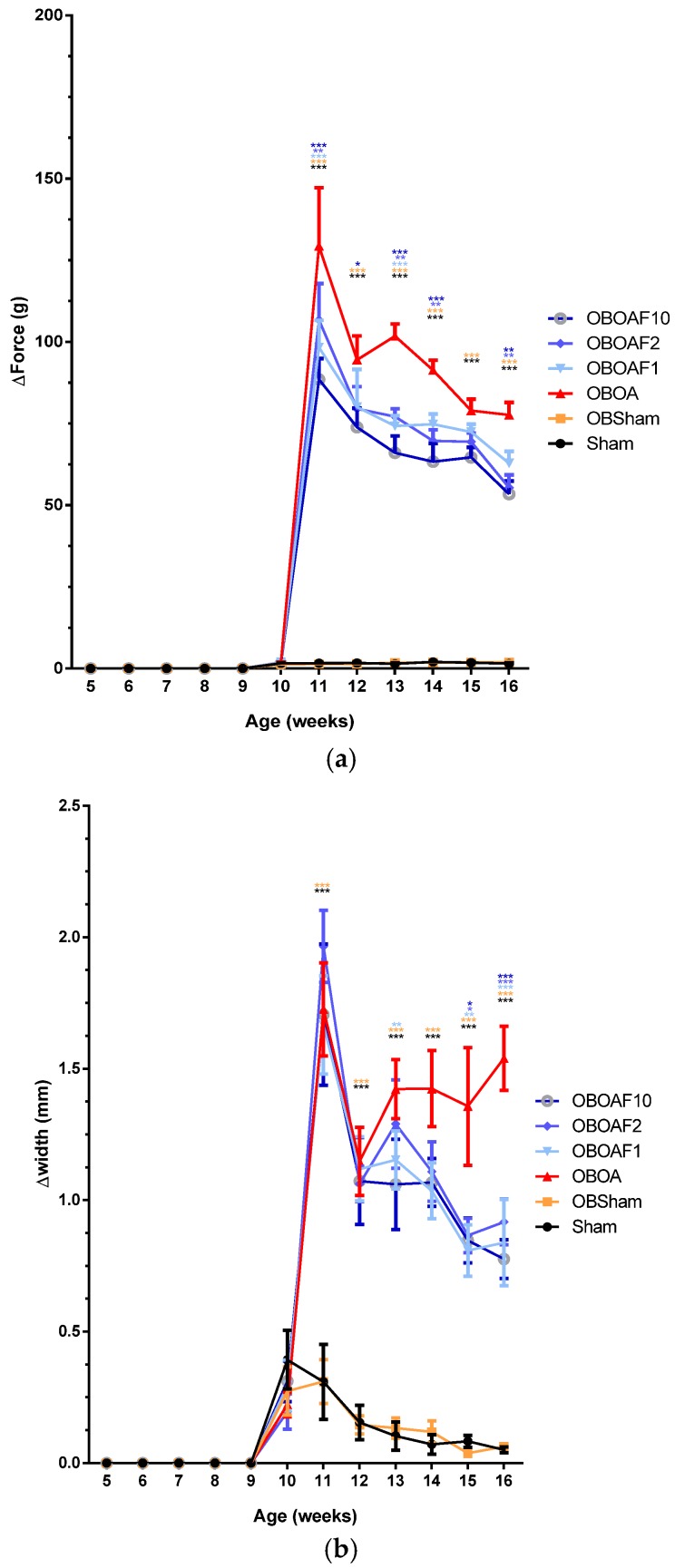
The effect of fucoidan treatment in HFD-induced obese male rats with ACLT + MMx surgery induced OA. (**a**) The on the weight-bearing distribution of the hind limbs. (**b**) The knee joint width. The data are the difference between the weights applied to the contralateral and ipsilateral limbs, expressed as the mean ± S.E.M. A two-way ANOVA and Dunnett’s multiple comparisons test were used to analyze the data. * *p* < 0.05, ** *p* < 0.01, *** *p* < 0.001, when compared to the OA group.

Oral administration of fucoidan helps to alleviate the pain induced by OA, as shown by the diminishing of the hind limb force differences (Figure 6a). Post-surgery of OA results in the swelling of the joints. By the measurement of the knee width, it was ascertained that the joint that underwent surgery had joint swelling after the surgery and recovered within 2 weeks. Due to the inflammation caused by OA, the joint that underwent ACLT + MMx had swelled for a longer period of time. The treatment with fucoidan helped to alleviate the swelling as the knee width differences between both hind limbs diminished over time (Figure 6b).

## 4. Discussion

Being overweight and obesity acts as one of the risk factors in OA progression [10,36]. The overload effect on joint cartilage may explain part of the increased risk of osteoarthritis, especially for the osteoarthritis of the knee [12]. The reduction of the body weight is a strategy for OA treatment due to the reduction of joint loading or the mechanical force on the knees [37,38]. The metabolic/obesity phenotype shows increased loading on the weight-bearing joints and the increased importance of the role of adipokines on the development of OA [7]. In animals with obesity, there is a huge increase in white fat (adipose tissue) deposits due to the hyperplasia and hypertrophy of their adipocytes [39]. The oral administration of fucoidan reduced the body weight in the HFD-induced obese rats. In addition, the fucoidan supplements decreased the adipose tissue weight such as the perirenal and epididymal fat tissues (Table 1). Furthermore, the fucoidan administration reduced the triglyceride (TG), total cholesterol (TC), and LDL-Cholesterol levels (Table 2). The obesity condition was associated with the increase of plasma TG, TC, and the LDL-Cholesterol levels. In particular, the triglyceride and cholesterol levels are closely related to cardiovascular disorders [40,41,42]. The previous study showed that fucoidan decreased the body weight of the HFD-induced obese mice and reduced the epididymal fat tissue [29]. There was also a reduction in the plasma level of the TG, TC, and LDL-Cholesterol levels in mice fed with fucoidan.

Various pathological processes such as diabetes, obesity, cardiovascular disease, and atherogenic processes were associated with oxidative stress [43]. Antioxidant sources can be depleted and the activity of enzymes such as superoxide dismutase (SOD) and catalase can decrease when obesity persists for a long time (CAT) [44]. In individuals with obesity, the activity of SOD and glutathione peroxidase (GPx), which is significantly lower in obese persons compared to healthy persons, have an involvement in the progression of obesity-related health problems [45]. In addition, higher levels of malondialdehyde (MDA) are found in obese subjects compared to normal-weight subjects [46,47]. The determination of MDA was used for the monitoring of lipid peroxidation in the biological samples [48]. Supplementation with antioxidants would reduce the risk of complications related to obesity and oxidative stress [49]. The results of this study showed that fucoidan increased the SOD activity and reduced the malondialdehyde (MDA) level (Figure 2). The previous studies reported that fucoidan extracted from *Undaria pinnatifida* and *Sargassum bideri* showed potent antioxidant activity with a high inhibition of free radicals [26,27]. 

The elevation of obesity-associated oxidative stress is possibly due to the presence of immoderate adipose tissue. Because adipocytes and preadipocytes have been identified as a source of pro-inflammatory cytokines including IL-1, TNF-α, and IL-6, as well as adipokines such as leptin, adiponectin, resistin, and visfatin; they reflect the state of chronic inflammation [12,44]. Inflammatory cytokines TNF-α and IL-1 may stimulate the mitogen-activated protein kinase (MAPK) pathway and the p38/c-Jun N-terminal kinase (JNK) pathway to synthesize matrix metalloproteinase-1 (MMP-1), MMP-3, and MMP-13 [50,51]. Additionally, the aforementioned cytokines, combined with leptin, will stimulate the Janus kinase 2 (JAK2) pathway and induce nitric oxide synthase (NOS) II and produce nitric oxide (NO). Nitric oxide produced in joints may cause cartilage degradation and chondrocyte apoptosis [52]. On the other hand, leptin regulates chondrocyte proliferation and differentiation [53]. Excessive leptin exposure might stimulate the differentiation of chondrocytes and the formation of osteophytes [54,55]. In addition, it may be involved in OA progression in serum and synovial fluid by the regulation of pro-inflammatory markers such as IL-1β, IL-6, IP-10, and leptin in the obesity condition [21].

Under stained observation (Figure 4 and Figure 5), rats supplemented with fucoidan showed the reduction in cartilage thickness and protected the matrix articular cartilage degeneration from the erosion and denudation of hyaline cartilage to the mild abrasion with the retention of the articular surface layer. The cartilage degeneration is caused by the overexpression of matrix metalloproteinases (MMPs). The overexpression of IL-1β and TNF-α stimulates the production of MMP-1 [4,52,56]. In the present study, the fucoidan-treatment suppressed the expression of IL-1β and TNF-α. Thus, we hypothesized that fucoidan also suppressed the expression of MMP-1 at the articular surface and inhibited cartilage degeneration.

Osteoarthritis, in many cases, causes joint swelling, pain, and disability [5,6]. The pain is caused by the imbalance between the ipsilateral with contralateral limbs (weight-bearing imbalance) and results in a change in their posture. In addition, in molecular inflammation, prostaglandin E_2_ (PGE_2_) is connected in all the processes leading to the signs of inflammation such as pain, redness, and swelling [57,58]. In the OA cartilage, the IL-1β and TNF signaling mediated by the transcription factors NF-KB and AP-1 results in the autocrine production of these cytokines, as well as the expression of other inflammatory and chrondrolytic mediators including prostaglandin E_2_ [52]. In the present study, the weight-bearing test showed that rats induced by OA surgeries have higher differences in the force applied by both hind limbs. On the other hand, the oral administration of fucoidan protected the weight-bearing in HFD-induced rats with ALCT+MMx-induced OA. Lee et al. [6] reported that fucoidan showed protective effects on the monosodium iodoacetate (MIA) induced OA rats. Joint swelling is one clinical feature of OA assigned to inflammation and reflects the presence of synovitis due to the thickening or effusion of the synovium [59]. The fucoidan treatment reduces the swelling of the joint with the lower knee joint width compared with the non-treated OA rats (Figure 6).

Fucoidan has been studied for its bioactivities and shows benefits because of its anticoagulation [60,61], anti-inflammatory [62,63], hypolipidemic [29,64], and immunomodulatory properties [65,66]. The previous study investigated the anti-inflammatory effect of fucoidan on collagen-induced arthritis. In this study, the results suggested that the lower molecular weight of fucoidan works better in lowering inflammation [30]. In addition, fucoidan extracted from brown seaweed can decrease the pro-inflammatory cytokines such as IL-6 and TNF-α, whereas it can also enhance the activation of NK cells and T cells [67]. Previous studies showed that fucoidan can suppress the CCL22 production in M2 macrophages via NF-κB-depend transcription [68] and the promotion of an antigen-specific T cell immune response [69].

The osteoarthritis model was typically divided into primary osteoarthritis (naturally occurring phenomenon) and secondary OA (normally associated with causes and/or risk factors leading to OA in the joint). Secondary OA includes trauma and other diseases or disorders of the metabolism or the bone [7]. In this study, we used HFD with ACLT + MMx to mimic the joint injury caused by obesity with the results showing an increased mechanical force in the joint, especially in the knee joint and the post-traumatic induction of OA. Previous studies reported that obesity is associated with the onset and progression of knee OA or musculoskeletal complications [21,22,70]. In addition, obesity models from high fat/high sucrose diets is an independent risk factor for the onset of osteoarthritis in the anterior cruciate ligament-transected knee of rats [20]. Others model such as the iodoacetic acid induction method might be able to mimic OA in a short time. These models, however, are more similar to chemical-induced chondrocyte death rather than OA models [71]. Due to the additional weight applied on both hind limbs, the effect of ACLT + MMx induced OA would be more significant. In the case of obesity, we also measure the level of inflammatory cytokines in their circulatory system.

## 5. Conclusions

Fucoidan extracted from *Cladosiphon okamuranus* showed anti-inflammatory effects on HFD induced inflammation. The hypolipidemic properties worked against fat accumulation and protected the joint and cartilage on HFD induced male rats with ACLT + MMx surgery induced OA. In addition, supplementation with fucoidan decreased the leptin and IL-1β levels. Our results suggested that the oral administration of fucoidan may improve the obesity and meniscal/ligamentous injury-induced osteoarthritis.

## Figures and Tables

**Table 1:** The body weight and adipose tissue weight of high-fat diet-induced obese male rats that have an anterior cruciate ligament transection and a partial medial meniscectomy (ACLT + MMx) surgery induced OA.

Group	Sham	Obese	Obese + OA			
			Control	F1	F2	F10
			**Body Weight (g)**			
Initial	136.24 ± 1.58 ^a^	139.15 ± 2.98 ^a^	141.82 ± 4.61 ^a^	137.71 ± 1.43 ^a^	135.19 ± 2.34 ^a^	140.93 ± 2.83 ^a^
Final	385.47 ± 16.50 ^c^	530.89 ± 33.53 ^a^	537.94 ± 36.55 ^a^	477.98 ± 19.75 ^b^	489.45 ± 22.23 ^b^	477.61 ± 35.41 ^b^
			**Adipose Tissue Weight (g/100 g Body Weight)**			
Perirenal	1.54 ± 0.15 ^c^	3.23 ± 0.54 ^a^	2.54 ± 0.37 ^b^	2.23 ± 0.35 ^b^	2.17 ± 0.33 ^b^	2.05 ± 0.43 ^b,c^
Epididymal	1.02 ± 0.09 ^c^	2.21 ± 0.33 ^a^	2.06 ± 0.27 ^a,b^	1.80 ± 0.12 ^b^	1.85 ± 0.27 ^a,b^	1.87 ± 0.36 ^a,b^

The data are expressed as mean ± S.E.M (*n* = 7). The values with different superscript letters (a–c) represent significant differences (*p* < 0.05) via one-way ANOVA followed by Duncan’s multiple range test.

**Table 2:** The plasma lipid in high-fat diet-induced obese male rats that have an anterior cruciate ligament transection and a partial medial meniscectomy (ACLT + MMx) surgery induced OA.

Group	Sham	Obese	Obese + OA			
			Control	F1	F2	F10
TG	70.83 ± 3.37 ^b^	81.59 ± 4.61 ^a,b^	95.26 ± 8.04 ^a^	79.94 ± 5.68 ^a,b^	77.08 ± 7.19 ^a^	70.30 ± 3.57 ^a^
TC	89.43 ± 7.83 ^b^	120.13 ± 9.92 ^a^	125.81 ± 7.08 ^a^	98.29 ± 4.91 ^b^	91.52 ± 3.45 ^b^	88.57 ± 3.16 ^b^
HDL-C	39.23 ± 1.66 ^a^	41.44 ± 2.98 ^a^	39.69 ± 2.02 ^a^	41.86 ± 2.52 ^a^	38.65 ± 2.51 ^a^	38.60 ± 3.16 ^a^
LDL-C	36.03 ± 7.70 ^b^	62.38 ± 9.84 ^a^	67.07 ± 6.90 ^a^	40.45 ± 6.76 ^b^	37.46 ± 4.17 ^b^	35.91 ± 2.23 ^b^

Triglycerides: TG, Total cholesterol: TC, High-density lipoprotein-cholesterol: HDL-C, Low-density lipoprotein-cholesterol: LDL-C. The data are expressed as mean ± S.E.M (*n* = 7). The values with different superscript letters (a,b) represent significant differences (*p* < 0.05) via one-way ANOVA followed by Duncan’s multiple range test.
